# DPRP: a database of phenotype-specific regulatory programs derived from transcription factor binding data

**DOI:** 10.1093/nar/gkt1254

**Published:** 2013-12-02

**Authors:** David T. W. Tzeng, Yu-Ting Tseng, Matthew Ung, I-En Liao, Chun-Chi Liu, Chao Cheng

**Affiliations:** ^1^Institute of Genomics and Bioinformatics, National Chung Hsing University, Taichung 402, Taiwan, ^2^Department of Computer Science and Engineering, National Chung Hsing University, Taichung 402, Taiwan, ^3^Department of Genetics, Geisel School of Medicine at Dartmouth, Hanover, NH, USA, ^4^Agricultural Biotechnology Center, National Chung Hsing University, Taichung 402, Taiwan, ^5^Institute for Quantitative Biomedical Sciences, Geisel School of Medicine at Dartmouth, Lebanon, NH, USA and ^6^Norris Cotton Cancer Center, Geisel School of Medicine at Dartmouth, Lebanon, NH, USA

## Abstract

Gene expression profiling has been extensively used in the past decades, resulting in an enormous amount of expression data available in public databases. These data sets are informative in elucidating transcriptional regulation of genes underlying various biological and clinical conditions. However, it is usually difficult to identify transcription factors (TFs) responsible for gene expression changes directly from their own expression, as TF activity is often regulated at the posttranscriptional level. In recent years, technical advances have made it possible to systematically determine the target genes of TFs by ChIP-seq experiments. To identify the regulatory programs underlying gene expression profiles, we constructed a database of phenotype-specific regulatory programs (DPRP, http://syslab.nchu.edu.tw/DPRP/) derived from the integrative analysis of TF binding data and gene expression data. DPRP provides three methods: the Fisher’s Exact Test, the Kolmogorov–Smirnov test and the BASE algorithm to facilitate the application of gene expression data for generating new hypotheses on transcriptional regulatory programs in biological and clinical studies.

## INTRODUCTION

In the past decade, gene expression profiling by microarray and more recently by RNA-seq experiments has been extensively used to study transcriptional regulation, resulting in a plethora of expression data available in public databases such as the Gene Expression Omnibus (1). These data sets are informative in elucidating transcriptional regulation under various biological and clinical conditions. For example, a comparison of gene expression between breast cancer and normal breast tissues identifies differentially expressed genes (DEGs) that are presumably critical for carcinogenesis. Such gene expression alterations in response to conditional changes are programmed by a set of transcription factors (TFs). Unfortunately, TF activity is often regulated by phosphorylation/dephosphorylation and other posttranscriptional mechanisms and can be modified by mutation. Thus, it is usually difficult to identify TFs responsible for gene expression changes solely based on the expression of TFs (2–4).

In principle, the activity of TFs can be reflected by the expression changes of their target genes: on TF activation, the expression of a TF’s target genes are more likely to be upregulated in the case of a transcriptional activator, and downregulated in the case of a transcriptional repressor; the opposite would be expected if a TF is deactivated. For example, we cannot consistently detect the expression change of the malfunctional p53 with a point mutation that abolishes the tumor suppressor’s transcriptional regulatory activity in tumor samples. However, the p53 gene targets are more likely to be differentially expressed in the tumor sample with respect to a normal sample. Based on this rationale, several methods have been proposed to infer the regulatory activity of TFs based on the expression change of their target genes (5–7). These methods have achieved substantial success in yeast because the target genes for most yeast TFs have been determined by ChIP-chip experiments (8). Regardless, the application of these methods in human is limited by incomplete knowledge of TF–gene interactions. In fact, to systematically identify human TF–gene interactions, previous studies have attempted to predict TF targets based on the existing TF binding motifs in DNA regions upstream of genes, albeit with a high false-positive rate (5,9).

In recent years, technical advances have made it possible to systematically determine the target genes of TFs by ChIP-chip and ChIP-seq experiments (10,11). In fact, a large number of ChIP-chip and ChIP-seq data have been generated by large-scale projects or individual laboratories. For instance, the ENCODE consortium (Encyclopedia of DNA Elements) has generated 424 ChIP-seq profiles, including >120 human TFs with various cell lines (12). Additionally, enormous amounts of gene expression data have accumulated over the past decade from studies addressing biological and clinical questions. The increasing availability of ChIP-seq data sets provides us with an unprecedented opportunity to reanalyze these gene expression data to further understand and dissect the regulatory networks underlying these expression profiles. In previous reports, the ChEA databases collected large-scale ChIP-seq data and provided the integrative analysis of both ChIP-seq and gene expression data (13); the ChIP-array web server can integrate ChIP-seq data and gene expression profiles to construct regulatory networks (14). Both databases only support Fisher’s Exact test but database of phenotype-specific regulatory program (DPRP) provided three algorithms, suggesting better functionality and flexibility.

In this study, we established a second-level database, named the Database of Phenotype-specific Regulatory Programs, to facilitate the search and application of gene expression data for generating new hypotheses on transcriptional regulatory programs under diverse biological and clinical contexts. In the database, we have collected 984 gene expression data sets, which include 29 744 samples. Each data set has several phenotype-specific subsets, and each subset is a group of samples. To study DEGs between two subsets, we defined the subset pair as two subsets within a data set. In the database, we have collected 984 gene expression data sets, which include 29 744 samples and 3754 subset pairs. It contains a wide range of phenotypes such as disease, drug treatment and tissue type. Meanwhile, we have defined a collection TF–gene regulatory relationships containing 424 TF binding profiles derived from the ENCODE ChIP-seq data. We applied three different methods, the Fisher’s exact test, the Kolmogorov–Smirnov test (KS test) and the Binding Association with Sorted Expression (BASE) algorithm we previously developed (7), to integrate gene expression profiles and the TF–gene interaction data to infer regulatory networks underlying each expression data set. DPRP provides a user-friendly interface for generating testable hypotheses on transcriptional regulation underlying a wide range of biological and clinical phenomena. DPRP is freely available at http://syslab.nchu.edu.tw/DPRP/.

## MATERIALS AND METHODS

### Database construction

#### Gene expression data

We collected 984 gene expression data sets, which include 29 744 samples. These data sets were originally generated to explore differential gene expressions under various conditions or treatments, e.g. gene expression changes during development; differential gene expression between different subtypes of breast cancer. Thus, each data set has several phenotype-specific subsets and each subset has a group of samples. To identify DEGs for each data set, we selected the subsets with at least three samples, and then performed t-test between each pair of subsets without overlapping samples. We obtained the DEGs (significantly upregulated or downregulated genes) for 3754 subset pairs representing a wide range of biological contexts (Supplementary Table S1).

#### Phenotype annotation of gene expression data

To systematically annotate gene expression data and address synonymous issues, we used the Unified Medical Language System (UMLS) technology that provides a comprehensive catalog of medical concepts (15). The UMLS includes Metathesaurus, semantic network and lexical resources. To concentrate on human disease study, we limited the UMLS concepts to three disease-related semantic types: ‘Pathologic Function’, ‘Injury or Poisoning’ and ‘Anatomical Abnormality’. To obtain the UMLS concepts for each data set, we used the UMLS natural language processing tool, MetaMap program (15), to process the summary description and the Medical Subject Headings of the PubMed record of the data set. It resulted in 4162 data set-concept relations including 757 distinct UMLS concepts (Supplementary Table S2). The phenotype annotation facilitates users to search specific biological or clinical concepts in enormous gene expression data.

#### ChIP-seq data

We downloaded 424 ChIP-seq track files from the ENCODE project (16), which represent the binding profiles of >120 human TFs in different cell lines. Based on ChIP-seq data, we applied a method called Target identification from profiles (TIP) algorithm (17) to calculate the binding affinity of each TF with all human RefSeq genes (18), resulting in a matrix containing binding affinities for all TF–gene pairs. TIP is a probabilistic model for the identification of TF target genes. Moreover, TIP calculated the *P* value and the Q value for each TF–gene pair, allowing us to define the target gene set for each TF profile (a TF under a specific cell line).

### Inference of phenotype-specific regulatory programs

We applied three different methods to integrate gene expression data with TF binding data to infer the regulatory programs underlying expression profiles. Given a subset pair (e.g. estrogen treated versus untreated MCF7 cell lines), we inferred the regulatory programs responsible for the DEGs. We connected the significant TFs based on ChIP-seq data to construct a regulatory network, in which the TF→TF interactions were identified by the TIP algorithm (*P* < 0.01) and indicated that one regulates the transcription of the other. A brief description of these methods is as follows:

#### Fisher’s exact test

Given a subset pair, we select the upregulated and the downregulated DEGs with *P* < 0.01. In case that the number of DEGs with *P* < 0.01 is <500, we instead select the top 500 significant genes to ensure enough genes are included for stable results in subsequent statistical analyses. To estimate the significance of differential TF activity, we performed Fisher’s exact tests to examine the overlap between the up-/downregulated gene set and TF target genes. This method requires two cutoff values: one is used to define the up- and the downregulated genes, and the other is used to define the TF target genes. A more detailed description of applying Fisher’s exact test for TF activity inference can be found in previous studies (5,19).

#### KS test

Given a subset pair, we calculated the t-scores for all genes by comparing their expression levels between the two subsets. For each TF we performed KS test to compare the distributions of the t-scores between target genes and nontarget genes. To define the target gene set of a TF, we set the cutoff value as *P* < 0.01. If the number of target genes with *P* < 0.01 is <500, we select the top 500 significant target genes for the regulatory program analysis. For each TF, the KS test resulted in a *P* value, indicating the significance of its activity change, and a D value, indicating the direction of its activity change. A positive D value indicates that target genes of a TF have significantly higher expression levels than nontarget genes, and a negative D-value indicates the reverse. A similar KS test-based method has been proposed by Tsai *et al.* (6) to identify cell cycle–related TFs in yeast.

#### BASE algorithm

The cutoff values for defining TF target genes and DEGs are usually arbitrary and hard to determine in advance. Comparing with the Fisher’s exact test and the KS-test, BASE is a nonparametric algorithm that requires no cutoff setting for TF target genes or DEGs (20). First, we calculated the t-scores for all genes by comparing their expression levels between a pair of subsets, and sorted them in the decreasing order to obtain a ranked gene list. Each gene in the list is associated with a *t_i_*, the t-score for this gene, and a *b_i_*, the binding affinity of a TF to this gene calculated by TIP algorithm (17). Then we calculated a cumulative distribution function by aggregating 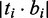 and a reference function by aggregating 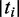. Finally, we calculated the maximum deviation between the functions and applied a permutation-based method to normalize the score and to estimate its significance. The normalized score is called regulatory activity score (RAS), which indicates the direction of the activity change of a TF. For a transcriptional activator, a positive/negative RAS indicates enhanced/reduced activity of the TF, while for a transcriptional repressor, the reverse is true. A more detailed description about BASE can be found in (20).

## WEB INTERFACES

**Figure 1.**
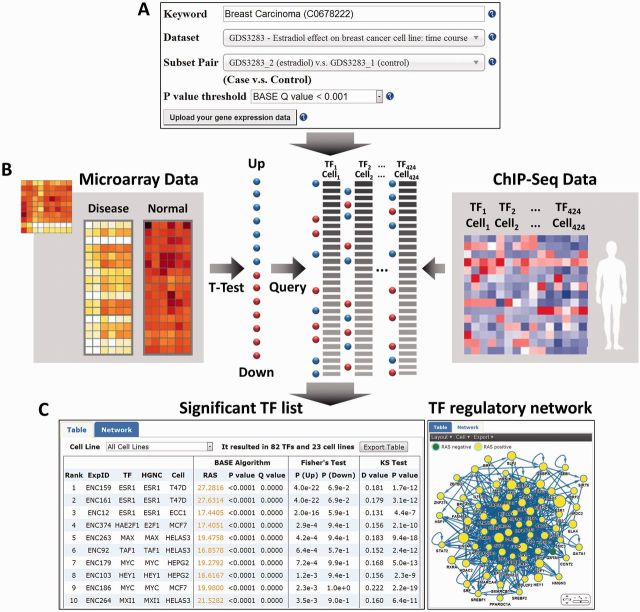
An overview of the DPRP web interface. (**A**) Users can perform a query by the following procedures: (i) Users can input a disease name in the auto-completed keyword field, which provides a list of partially matched UMLS concepts for selection. Alternatively, users can also input a data set ID in the keyword field to select a specific data set. (ii) After UMLS concept selection, the data sets associated with the selected concept will be shown in a data set list, from which the user can select the data set of interest. (iii) Given a specific data set, the subset pairs from the selected data set will be displayed in a subset list, and then the user can select the subset pair to search TF regulatory programs. DPRP provides three different methods to rank the potential TFs, in which users can determine which ranking guidelines to use. In addition, users can upload their own gene expression data with gene list and t-value of t-test or log ratios between two subsets. (**B**) The database integrated gene expression data and ChIP-seq TF binding data to identify the regulatory programs underlying a selected phenotype pair. (**C**) The output web pages: DPRP generates a list of the TFs and ranks them by their *P* values or Q values. In TF table view, users can export the table of candidate TFs as a text file. Based on the ranked TF list, DPRP generates a regulatory network consisting of all significant TFs, in which users can export the TF network as a png, svg or xml file.

We integrated gene expression data sets, phenotype information and ChIP-seq data sets to construct the DPRP database with a user-friendly web interface (Figure 1). Users can search a disease concept to discover all related gene expression data sets, choose the interested data set and then select a subset pair within the data set for TF regulatory program analysis. For example, a user can type in ‘Breast Carcinoma’ as a keyword to obtain a list of data sets related to breast cancer. To facilitate user-friendly text search, we adopted the jQuery AutoComplete technique to guide the user for keyword selection (http://jqueryui.com/autocomplete/). When a specific data set is selected, the database will list a number of subset pairs (e.g. breast cancer versus normal) for investigating regulatory activity of TFs.

Given a subset pair, DPRP will generate a list of TFs with significant activity changes. To visualize the TF regulatory program, the web server draws a regulatory TF network consisting of all significant TFs, in which the TF→TF interaction indicates that one regulates the transcription of the other, which is identified by the TIP algorithm (*P* < 0.01) from ChIP-seq data. The ChIP-seq data support TF-gene regulations in different cell lines. Users can select a specific cell line to display a cell line–specific network or use all cell lines to display an integrated network. Some cell lines only have a few ChIP-seq experiments, which is not sufficient for TF network construction. Thus, the web interface only allows users to select cell lines with at least 12 ChIP-seq experiments. Moreover, users can upload their own gene expression data onto the database, and then DPRP will perform Fisher’s Exact test, KS test and BASE algorithm in the data.

## EXAMPLE APPLICATIONS

**Figure 2.**
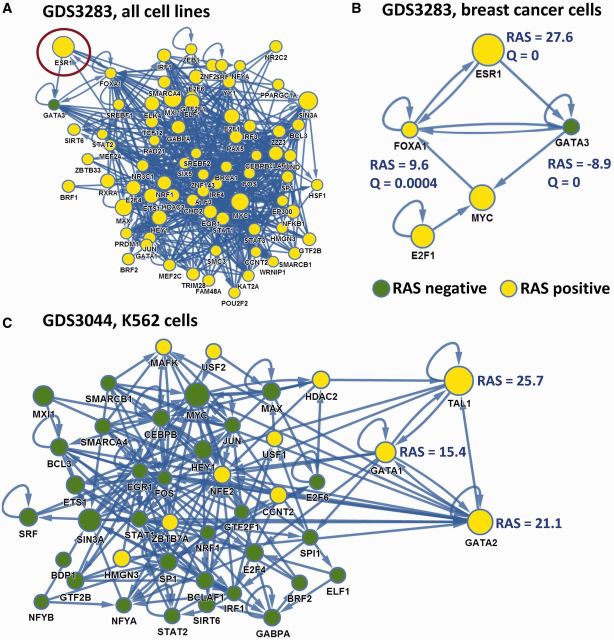
Example applications. (**A**) The complete regulatory TF network associated with estradiol treatment in MCF7 cells from the GDS3283 data set. The network contains 74 significant TFs identified from ChIP-seq data in all cell lines, in which the most significant TF is ESR1. This is the regulatory network output by the BASE method with Q < 0.001, when users select the GDS3283 data set and subset pair ‘estradiol treatment versus control’. (**B**) The regulatory TF network specific for T47D + MCF7 cell lines. In the network, only the significant TFs with ChIP-seq data from T47D and MCF7 are displayed. (**C**) The regulatory TF network associated with imatinib treatment in K562 cells from the GDS3044 data set. This is the output by the BASE method (Q < 0.001), when users select the GDS3044 data set with subset pair ‘imatinib treatment versus control’, and then select the K562-specific TF network. The network contains 43 significant TFs, in which the most significant TF is TAL1.

To demonstrate the biological importance of DPRP, we used GDS3283 and GDS3044 as examples to show the cell-specific regulatory TF networks (Figure 2). GDS3283 is a gene expression data set with estradiol treatment using MCF7 breast epithelial cancer cells (21). The BASE algorithm identifies 74 TFs with significantly differential activity (Q < 0.001), in which the most significant TF is estrogen receptor alpha (ESR1) based on ChIP-seq experiments carried out in T47D cells (Figure 2A). Obviously, this result is consistent with our knowledge that the estradiol treatment significantly induces the activity of ESR1 in breast cancer cells. Interestingly, when the expression levels of ESR1 are compared between estradiol treatment and control samples, we cannot detect significant expression change of ESR1 in GDS3283. Thus, the BASE algorithm identified the key regulator that cannot be discovered by differential expression analysis.

Since the GDS3283 is a breast cancer data set, we selected the ChIP-seq experiments from breast cancer cell lines (T47D and MCF7 cells) to generate the regulatory TF network with Q < 0.001 (Figure 2B), which contained five significant TFs: ESR1, GATA3, FOXA1, MYC and E2F1. In this regulatory network, ESR1, FOXA1 and GATA3 formed a tight regulatory module. These results are consistent with the finding by Kong *et al.* (22) that FOXA1 and GATA3 are essential co-regulators in estrogen response pathway and that ESR1, FOXA1 and GATA3 formed an enhanceosome in breast cancer cells. Activation of MYC and E2F1 may indicate that estrogen treatment can promote cell proliferation of MCF7 cells.

DPRP also provides insights into drug mechanisms. GDS3044 is a gene expression data set in K562 (the leukemia cell line) cells treated with imatinib. Figure 2C shows that the imatinib treatment significantly increases the activity of three TFs: GATA1, GATA2 and TAL1 in K562 cells. It is known that imatinib inhibits the kinase activity of BCR-ABL protein, which is the pathophysiologic cause of chronic myelogenous leukemia. Previous studies have shown that BCR-ABL suppresses the GATA1 activity, and thus explains why we observed an increased activity of GATA1 in response to imatinib treatment (16,23). In addition, TAL1, the T-cell acute lymphocytic leukemia protein 1, is specifically expressed in early erythroid cells and interacts with GATA1 (24), which also supports our result.

## DISCUSSION

In this study, we applied three different methods to infer the regulatory programs underlying given gene expression profiles. To apply the Fisher’s exact test, DEGs have to be defined based on the gene expression data. At the same significance level, the numbers of DEGs vary substantially in different gene expression data sets, depending on the quality of the data and the sample size. As a consequence, we expect variability in statistical power and robustness of Fisher’s exact test. Similarly, the effectiveness of this method is also influenced by the number of TF target genes. TFs with more target genes are more likely to be identified as significant TFs. The KS-test does not require the up-/downregulated gene sets, but it still requires the predefined TF target genes. The BASE method requires neither a differential gene set nor a target gene set, and thus is more convenient in practice and does not have the bias issue. However, it estimates the significance of the TF RAS using the permutation of gene expression profiles (shuffle all genes in the profile), which often overestimates their significance. Because none of these methods is perfect, we provide the results from all three methods in the DPRP database. This allows users to determine the stringency level and make decisions according to their own requirements, e.g. selecting the significant TFs identified by all methods to obtain a TF list of high confidence.

Currently, we have included the ChIP-seq data generated by the ENCODE project in our database. There are many ChIP-seq and ChIP-chip data sets that have been generated by other large-scale projects or by individual laboratories that will be included in the database. Moreover, we anticipate that an increasing number of TF ChIP-seq data will be generated in the near future. We will maintain our database with routine updates to ensure that we maintain a comprehensive list of TFs. We believe DPRP will be a useful database and resource for biological and clinical studies.

## SUPPLEMENTARY DATA

Supplementary Data are available at NAR Online.

## FUNDING

American Cancer Society Research [IRG-82-003-27]; start-up funding package provided by the Geisel School of Medicine at Dartmouth College (to C.C.); National Science Council grants [NSC99-2320-B-005-008-MY3 and NSC101-2627-B-005-002 to C.C.L.]. Funding for open access charge: American Cancer Society Research Grant, [#IRG-82-003-27].

*Conflict of interest statement*. None declared.
